# Supplementary material for: Early path dominance as a principle for neurodevelopment
Source: Proc Natl Acad Sci U S A. 2023 Apr 13;120(16):e2218007120. doi: 10.1073/pnas.2218007120 (PMC10120000; doi:10.1073/pnas.2218007120)
Supplement: Supplementary file 1 — Appendix 01 (PDF) [file pnas.2218007120.sapp.pdf]

Supplementary Information

Diffusion MRI data sets

United Kingdom Biobank

The United Kingdom (UK) Biobank is an ongoing epidemiological study aimed at discovering early markers for brain diseases [28]. UK Biobank is an excellent data source because of its large sample size (current release at our disposal: 19,380 people, ages 45 to 80 (mean age is 62.6 ± 7.4 years old)) and data standardization procedures – all corresponding measurements are collected on the same machines across several sites operating the same software [33]. More details on the minimally preprocessed dMRI images publicly assessed from UK Biobank can be found in Alfaro-Almagro et al. [33].

Individuals' brain networks under the Talairach atlas have 727.4 ± 2.6 nodes with an average degree of 28.3 ± 1.1 (prior to targeted attack). The Spearman correlation between tract length and density is -0.20 ± 0.02 (Figure S20).

Adolescent Brain Cognitive Development Study

The Adolescent Brain Cognitive Development (ABCD) Study is an ongoing longitudinal study of ten years focused on understanding brain development during the critical period of adolescence [29]. The ABCD Study complements the UK Biobank by having individuals with an age range of 8.9 to 13.3 (mean age is 10.6 ± 1.1 years old), for the current data release at our disposal of 15,593 people (release 4.0, NIMH Data Archive Digital Object Identifier 10.15154/1526472). More details on the minimally preprocessed dMRI images of the ABCD Study publicly assessed from the National Institute of Mental Health Data Archive can be found in Hagler et al. [61].

Individuals' brain networks under the Talairach atlas have 731.3 ± 2.6 nodes with an average degree of 31.0 ± 3.5 (prior to targeted attack). The Spearman correlation between tract length and density is -0.28 ± 0.05 (Figure S20).

Developing Human Connectome Project

The Developing Human Connectome Project (dHCP) acquires MRI from neonates within weeks of birth to probe early development [30]. The dHCP contains the youngest human subjects publicly accessible, gestational ages 26.7 to 45.1 weeks old (mean gestational age 39.7 ± 3.5 weeks old). We have 758 scans at our disposal from the current data release (third data release). More details on the minimally preprocessed dMRI images publicly assessed from dHCP can be found in Christiaens et al. [62].

Individuals' brain networks under the Talairach atlas have 733.4 ± 1.9 nodes with an average degree of 34.4 ± 2.6 (prior to targeted attack). The Spearman correlation between tract length and density is -0.27 ± 0.03 (Figure S20).

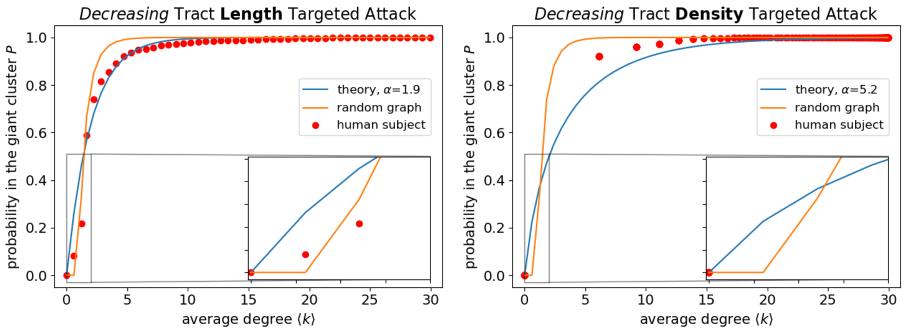

Figure S1: Targeted attack on *decreasing* tract lengths and tract densities. Note that throughout the main text, we focus on targeted attack of increasing tract lengths and densities and derive an analytical solution based on the Giant Cluster Self Preference theory (Equation 2), which is also shown in this figure for comparison. Targeted attack on decreasing tract lengths essentially follows a random graph. It is not possible to fully assess the corresponding P curve of tract density because of the large gap at low average degree. Curves are derived from dMRI data collected by the UK Biobank under the Talairach atlas for one human individual (subject ID: 6025360), the same subject as in Figure 2.

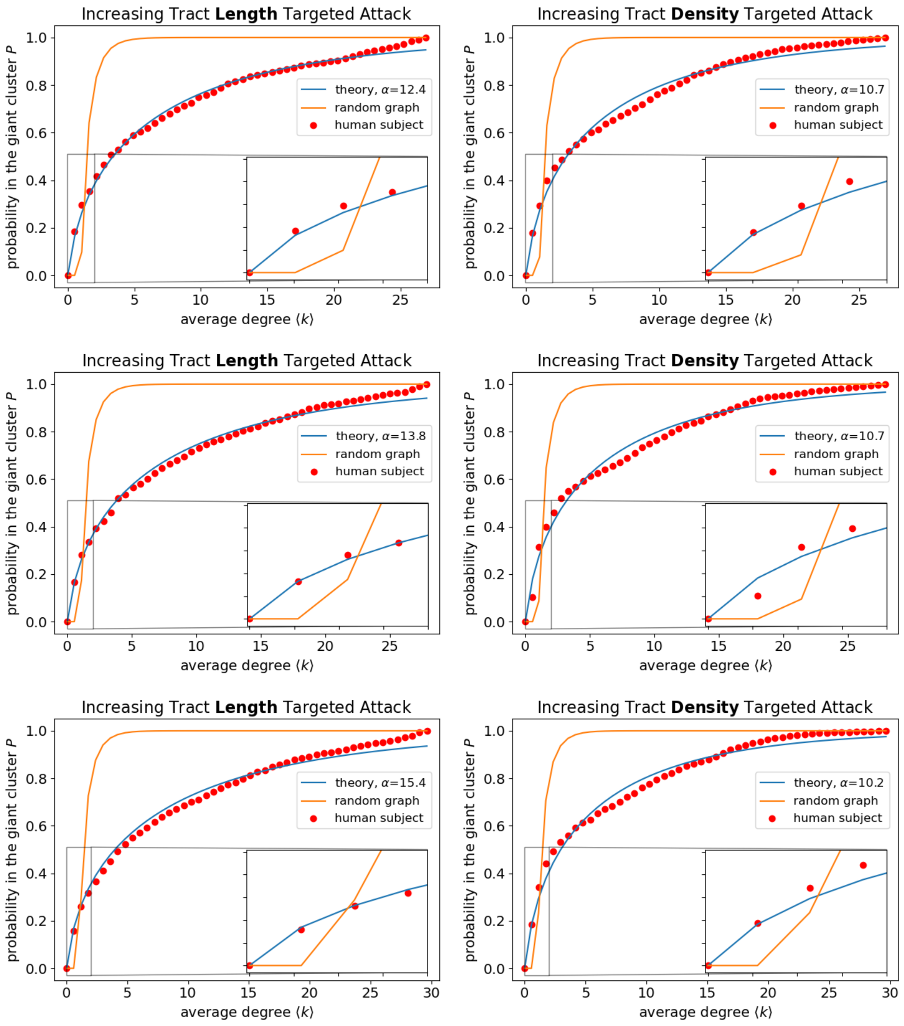

Figure S2: P curves for additional individuals besides that shown in Figure 2. From the top to bottom row, the UK Biobank subject IDs are 1701210, 1363111 and 2460658. The fitted α parameters are fairly similar among different subjects.

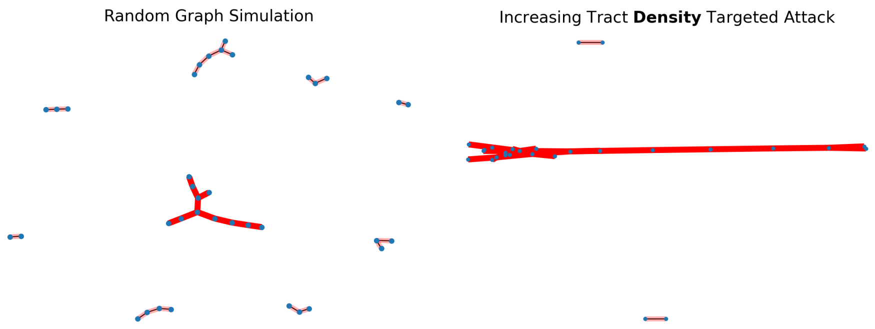

Figure S3: Visualizing cluster formation at average degree $\langle k \rangle \sim 1$ for both a random graph simulation (left) and the brain under increasing tract density targeted attack (right). The random graph simulation has several non-negligible secondary clusters, while the brain essentially has only one densely connected, large cluster. The random graph simulation has the same number of nodes as that of the brain shown analyzed under the Harvard-Oxford atlas, 64 total regions, however we exclude isolated nodes in both graphs for ease of visualization. The brain's cluster distribution is derived from dMRI data collected by the UK Biobank for one human individual (subject ID: 6025360), the same subject as in Figure 2, however, analyzed at a coarser parcellation to easily visualize clusters.

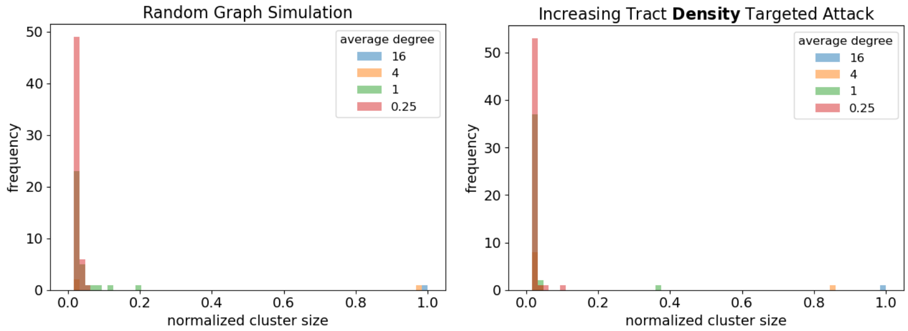

Figure S4: Cluster distributions across several average degrees for both a random graph simulation (left) and the brain under increasing tract density targeted attack (right). The random graph simulation has several non-negligible secondary clusters at small $\langle k \rangle$, while the brain essentially has only one densely connected, large cluster throughout. The random graph simulation has the same number of nodes as that of the brain shown analyzed under the Harvard-Oxford atlas, 64 total regions, as in Figure S3. Normalized cluster size is equal to cluster size divided by 64. Frequency refers to the number of different nodes with that cluster size. The brain's cluster distribution is derived from dMRI data collected by the UK Biobank for one human individual (subject ID: 6025360), the same subject as in Figure 2, however, analyzed at a coarser parcellation.

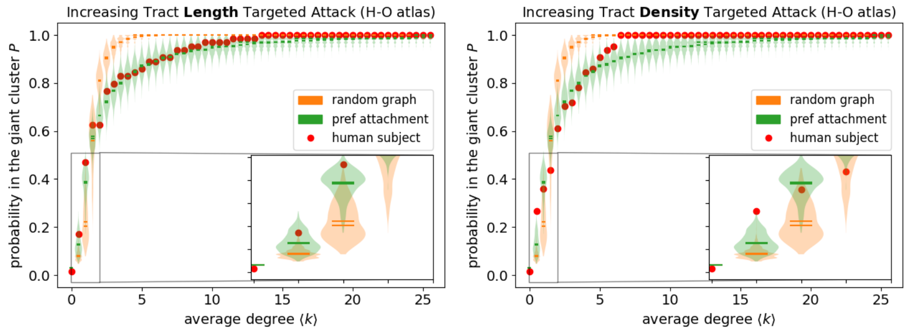

Figure S5: A network built by preferential attachment captures experimental P curves at a coarser parcellation, called the Harvard-Oxford (H-O) atlas. Networks are presented as violin plots of simulations (1,000 independent runs). Simulations have the same number of nodes as regions under the H-O atlas (64) as well as the same final average degree of 25.5 seen for the corresponding subject. Experimental curves are derived from dMRI data collected by the UK Biobank under the Harvard-Oxford atlas for one human individual (subject ID: 6025360), the same subject as in Figure 2.

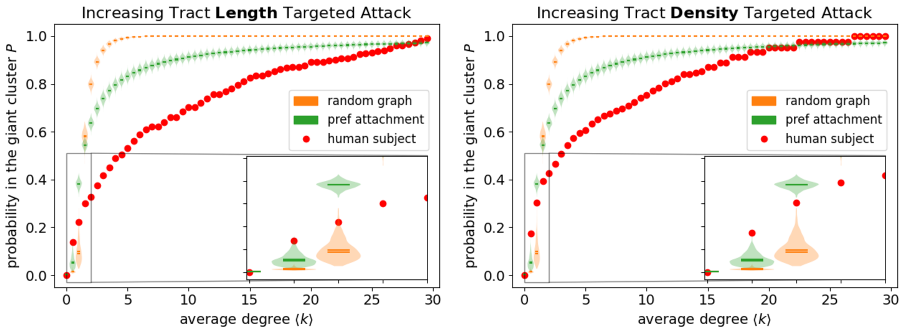

Figure S6: Random and preferential attachment networks are not able to capture P curves tabulated with the same number of nodes as regions under the Talairach atlas (727), as well as the same final average degree of 29.9. Violin plots for the networks are determined as in Figure S5. Experimental curves are derived from dMRI data collected by the UK Biobank under the Talairach atlas for one human individual (subject ID: 6025360), the same subject as in Figure 2.

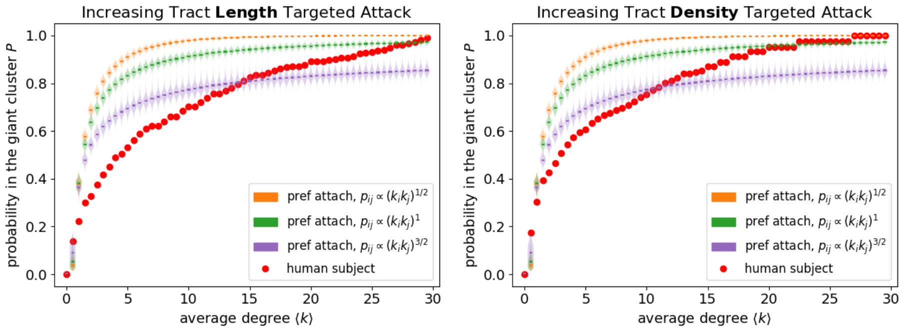

Figure S7: General preferential attachment networks in which the probability of edge formation p_{ij} is proportional to the multiple of nodes' i and j degrees $k_i * k_j$ to some power x [63, 64]. What is referred to as preferential attachment in the main text and in Figure S6, corresponds to $p_{ij} \propto k_i k_j$ in this figure. Despite considering general preferential attachment networks with $x = 1/2$ and $x = 3/2$, we are still not able to capture P curves tabulated with the same number of nodes as regions under the Talairach atlas (727), as well as the same final average degree of 29.9. Violin plots for the networks are determined as in Figure S5. Experimental curves are derived from dMRI data collected by the UK Biobank under the Talairach atlas for one human individual (subject ID: 6025360), the same subject as in Figure 2. Note that we are not able to explore arbitrarily large x because those simulation start sampling the same edges over and over, causing the simulation to freeze and not add any new edges.

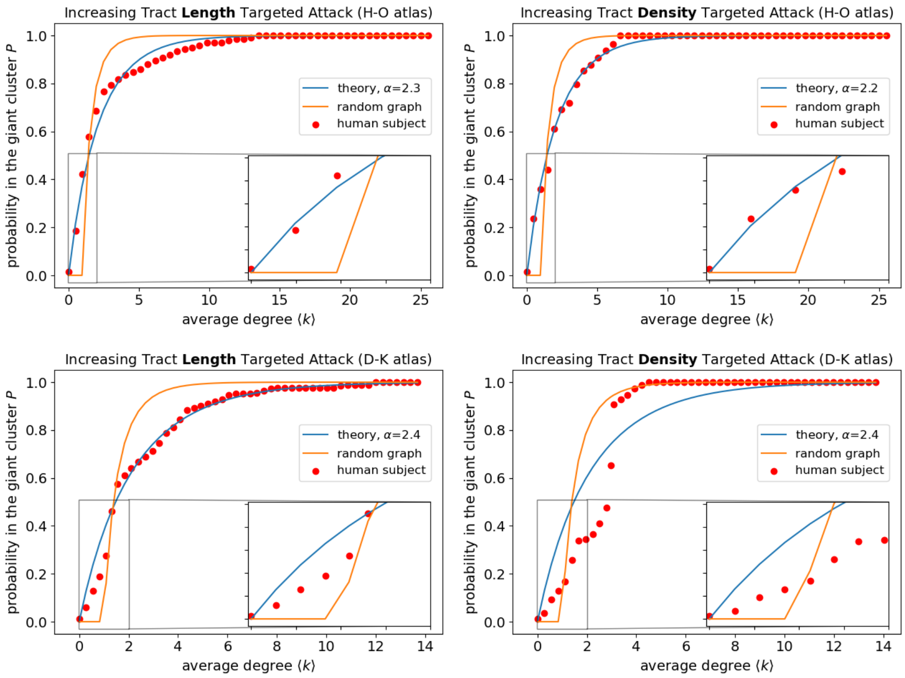

Figure S8: The Giant Cluster Self Preference theory mostly captures P curves generated from coarser atlases with smaller fitted α. Both atlases considered contain fewer regions than the Talairach atlas (727 regions) used throughout the main text; Harvard-Oxford (H-O) and the modified Desikan-Killiany (D-K) atlases have 64 and 84 regions considered in our analysis, respectively. Curves are derived from dMRI data collected by the UK Biobank for one human individual (subject ID: 6025360), the same subject as in Figure 2. It is not clear why tract density targeted attack under the D-K atlas deviates from the Giant Cluster Self Preference theory. The results look somewhat similar to a random graph and demonstrate a sharp rise in its P curve.

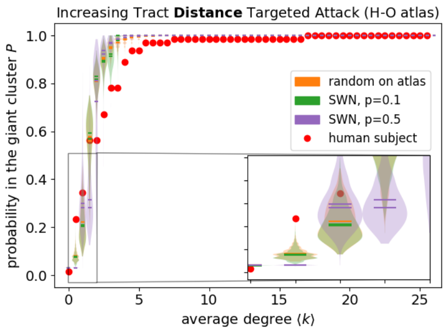

Figure S9: A random network based on actual coordinates of regions according to the Harvard-Oxford (H-O) atlas does not capture P curves. Small-world networks (SWNs) are also not able to capture P curves tabulated with the same number of nodes as regions under the Harvard-Oxford atlas. Networks are presented as violin plots of simulations (1,000 independent runs). Small-world networks are defined as in [1] and the parameter p in the legend corresponds to the probability of rewiring each edge. For both network types, we first constructed the final network with the correct number of nodes (64) and final average degree (25.5). We then performed targeted attack on increasing tract distance. Note that tract distance is more limited than tract length because it assumes that the trajectory of the white matter tract between the two regions is a straight line. Nonetheless, we find that targeted attack on increasing tract distance provides very similar results to that of tract length (Figure S15). The experimental curve is derived from dMRI data collected by the UK Biobank under the Harvard-Oxford atlas for one human individual (subject ID: 6025360), the same subject as in Figure 2.

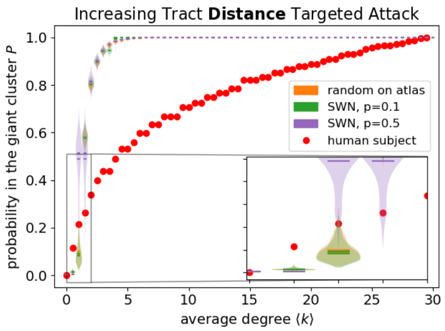

Figure S10: A random graph constrained by the atlas and small-world networks (SWNs) are not able to capture P curves tabulated with the same number of nodes as regions under the Talairach atlas (727), as well as the same final average degree of 29.9. Violin plots for the networks are determined as in Figure S9. Curves are derived from dMRI data collected by the UK Biobank under the Talairach atlas for one human individual (subject ID: 6025360), the same subject as in Figure 2.

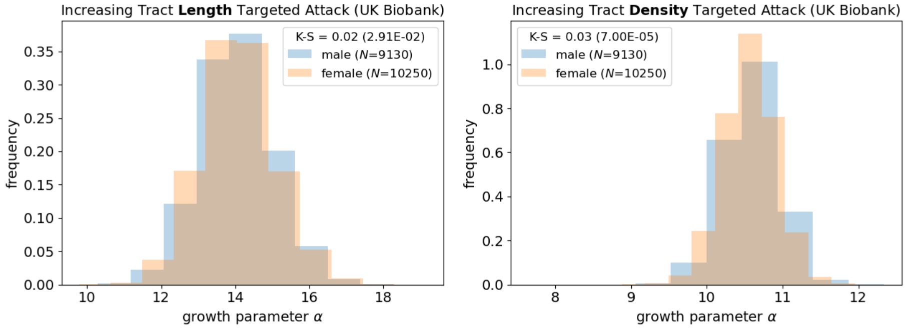

Figure S11: Distributions of the fitted α parameter does not vary much between individuals of different sexes. K-S corresponds to the Kolmogorov-Smirnov test statistic, with p-value in parentheses. The variable N represents the total number of individuals of the corresponding sex in the UK Biobank.

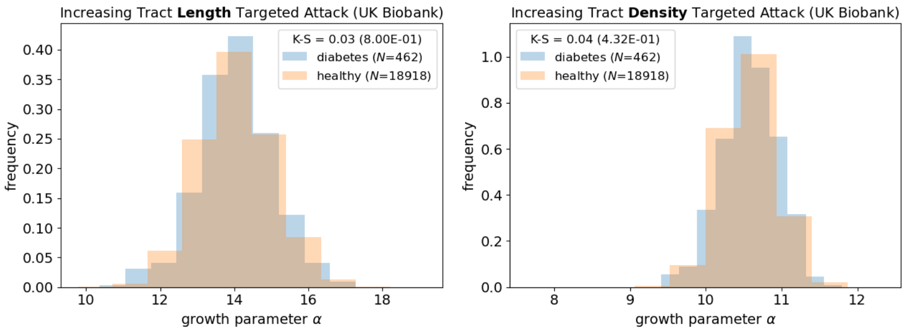

Figure S12: Distributions of the fitted α parameter does not systematically vary between individuals diagnosed with diabetes and those not diagnosed with diabetes (healthy). K-S corresponds to the Kolmogorov-Smirnov test statistic, with p-value in parentheses. The variable N represents the total number of individuals with the corresponding diabetes diagnosis in the UK Biobank.

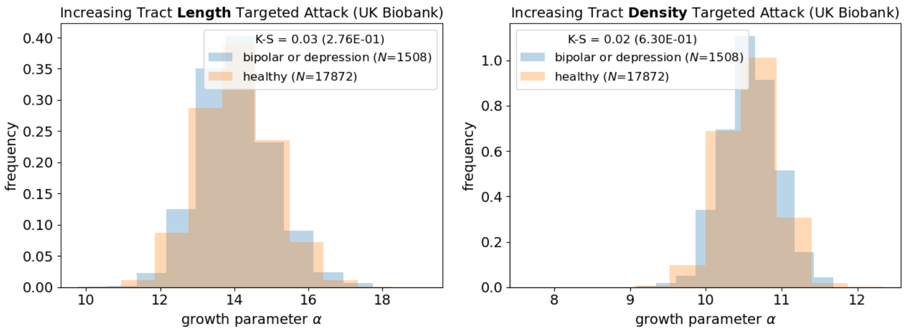

Figure S13: Distributions of the fitted α parameter does not systematically vary between individuals diagnosed with bipolar disorder or depression and those not diagnosed (healthy). K-S corresponds to the Kolmogorov-Smirnov test statistic, with p-value in parentheses. The variable N represents the total number of individuals with the corresponding bipolar disorder and depression diagnosis in the UK Biobank.

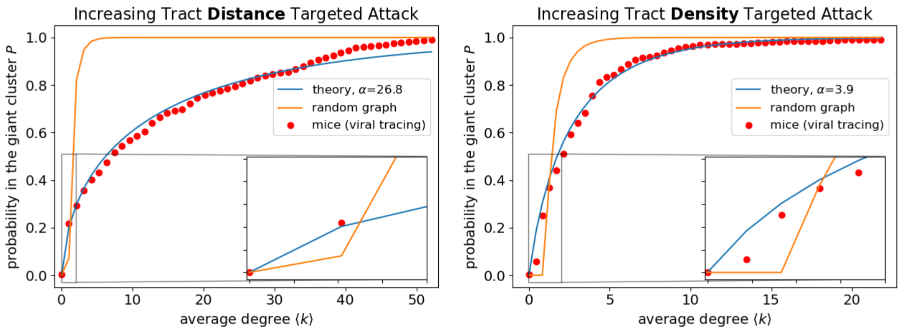

Figure S14: The Giant Cluster Self Preference theory captures the probability for a brain region to be in the giant cluster across average degree with an appropriately fitted α parameter for both targeted attack by increasing distance between regions' center of masses and tract density. Curves are derived from viral tracing data by the Allen Institute averaged over $\sim$1000 mice [31]. While α values for the two human P curves are comparable at 15.3 and 11.0 (Figure 2), the α value shown here needed to fit the distance P curve is around five times larger than that of the tract density for mouse. The relative differences do not seem to be a result of viral tracing being limited to distances; we find similar trends if we look at corresponding distances for human results (Figure S15). Also, the resolution of the atlas does not seem to be a factor; the atlas for the presented mouse results contains 426 grey matter regions, while that for human contains 727 grey matter regions. Changing the atlas used in processing the dMRI to calculate the connectivity matrix to 84 or 64 regions, yields α(distance)/α(density) even closer to 1 (Figure S8). Further investigation is needed to elucidate whether relative α differences are a result of differences between the dMRI and viral tracing data modalities as highlighted in other studies [65, 66].

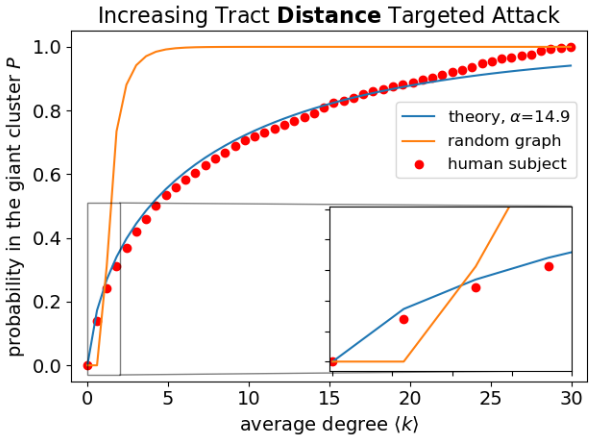

Figure S15: Targeted attack based on center of mass distances of Talairach atlas regions yields similar results as compared to tract lengths. Curves are derived from dMRI data collected by the UK Biobank under the Talairach atlas for one human individual (subject ID: 6025360), the same subject as in Figure 2.

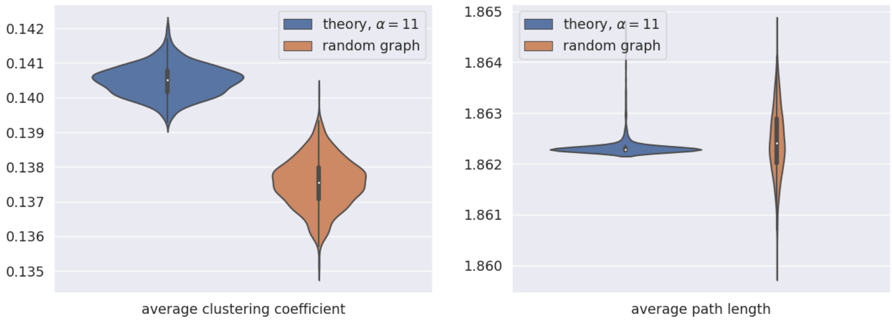

Figure S16: The Giant Cluster Self Preference theory slightly reproduces elevated average clustering coefficients, the hallmark of small-world networks. Our theory's average clustering coefficient is slightly larger than that of a random graph. In addition, our theory's average path length is essentially the same as that of a random graph, as predicted by a small-world network. Graph properties are calculated for 1000 independent runs and presented as violin plots with the same number of nodes and final average degree for both graph types. The number of nodes is set to 727, the same number as in the Talairach atlas, and the final average degree is set to 100 to ensure $P = 1$ at the end of each simulation run. Edges are added with $\alpha = 11$ according to the Giant Cluster Self Preference theory.

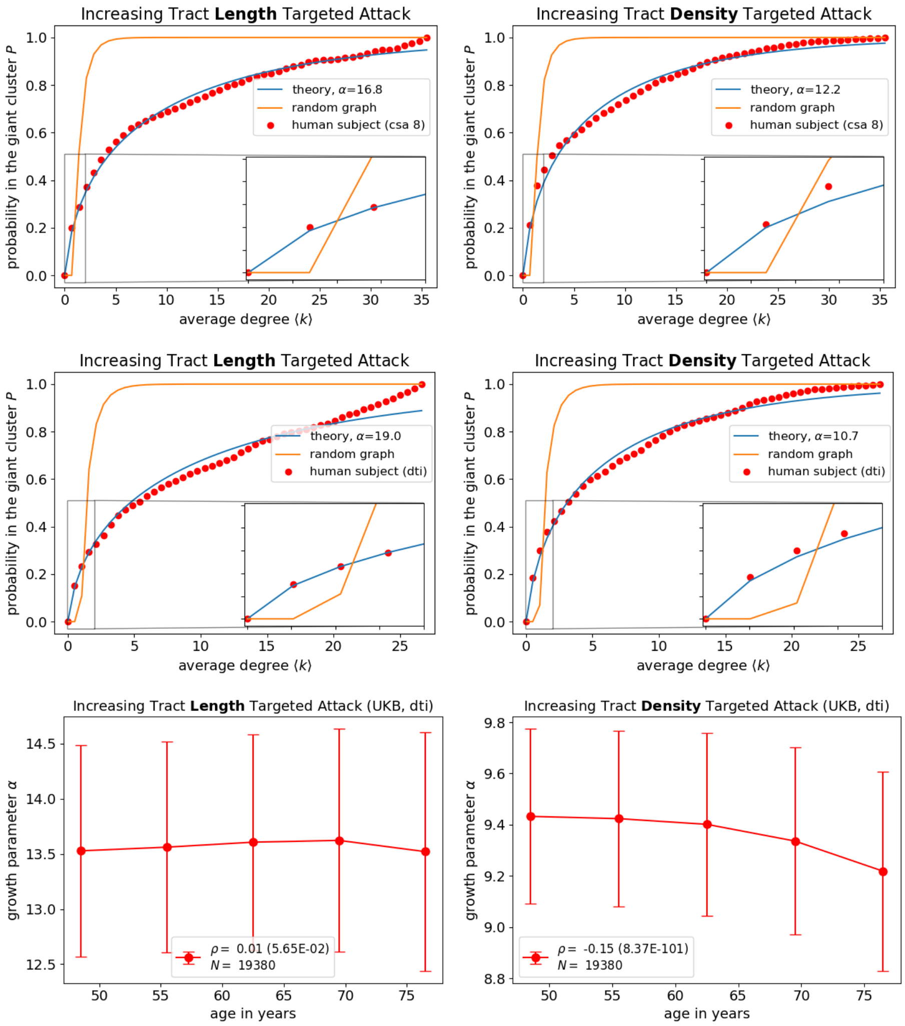

Figure S17: Changing the default procedure in DIPY to calculate the connectivity matrix from dMRI data have little effect on the P curves. In the top two figures, we alter the constant solid angle (csa) method to spherical harmonic order 8, compared to order 6 in the main text. In the middle two figures, we use the diffusion tensor imaging (dti) method, rather than the constant solid angle method used in the main text. Curves are derived from dMRI data collected by the UK Biobank under the Talairach atlas for one human individual (subject ID: 6025360), the same subject as in Figure 2. In the bottom two figures, we show results across the entire UK Biobank (UKB) using the dti method. Although increasing tract length targeted attack results are strikingly similar as using the default procedure (Figure 3), those for tract density have slightly lower α values that are more correlated with age.

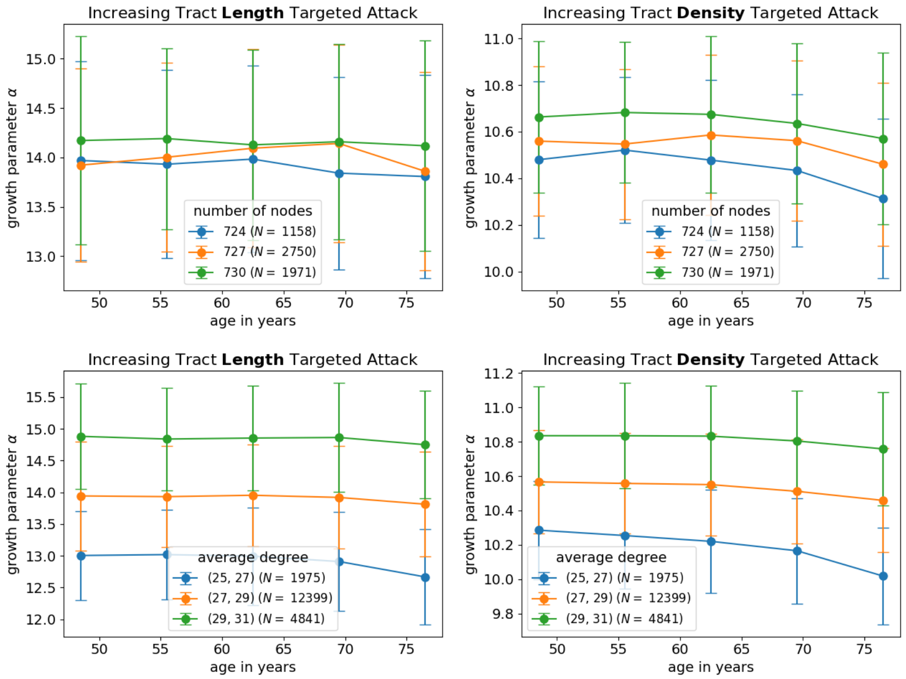

Figure S18: Values of α are fairly robust to different number of nodes and average degrees of brain networks in the UK Biobank. Binned data are presented with a line connecting means, and error bars correspond to standard deviations. Thresholds are chosen based on histograms of the respective property (Figure S20). Average degree thresholds encompass a range because values are not discrete. For example (25, 27) corresponds to all networks with average degrees larger than 25 but less than 27. Some variability in α is seen as network average degree changes, however, variability is systematic across age such that α values differ by a constant.

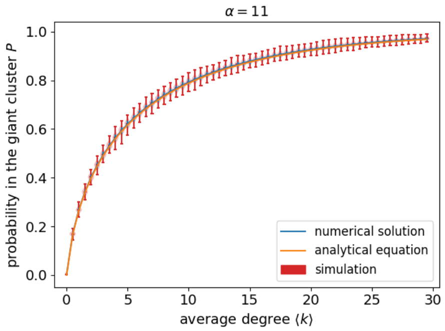

Figure S19: Simulation results verify our theoretical derivation of Equation 9. Violin plots represent 1000 independent runs of a graph with 727 nodes, the same number as in the Talairach atlas, and a final average degree of 100 to ensure that at the conclusion of the simulation, all runs have P=1. The probability that an edge is added between nodes in the giant cluster is set to $p = 100/727$, while the probability that an edge is added between a giant cluster node and a non-giant cluster node is $p/11$ because α is set to 11. The blue line corresponds to the numerical solution of Equation 5, with error bars representing one standard deviation for the respective $p(n|E)$ across n. The orange line corresponds to Equation 9 and approximately solves Equation 5 as discussed in the Methods. The analytical equation is simply referred to as 'theory' throughout other figures in the main text and SI.

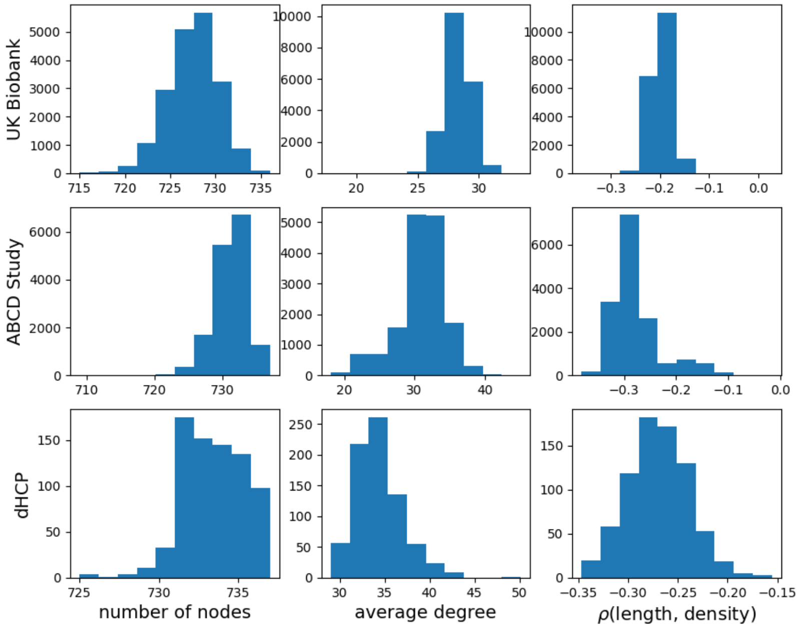

Figure S20: Histograms of basic network properties across individuals in the UK Biobank ($N = 19380$), ABCD Study ($N = 15593$) and dHCP ($N = 758$) data sets. The number of nodes, the average degree of the network before targeted attack is performed, and the Spearman correlation (ρ) between tract length and tract density are shown. The y-axis corresponds to the number of individuals with the respective network property value.
